# Physiological Chemistry

**Published:** 1856-07

**Authors:** 


ART. VII.—1. *Physiological Chemistry;* form the German of Prof. C. G. LEHMANN, M. D. Translated by GEORGE E. DAY, M. D., and Edited by Prof. R. E. ROGERS, of the University of Pennsylvania. 2 volumes, 8vo. Philadelphia: BLANCHARD & LEA. 1856.

2. *Manual of Chemical Physiology*; from the German of Prof. C. G. LEHMANN, M. D. Translated with Notes and Additions by J. CHESTON MORRIS, M. D. With an Introductory Essay on Vital Force, by SAMUEL JACKSON, M. D., Prof. of Institutes of Medicine in the University of Pennsylvania. Philadelphia: BLANCHARD & LEA. 1856.

The second of these works may be regarded as an abridged translation, with notes by Mr. Jackson, while the first is a full translation of Prof. Lehmann's second edition of his Physiological Chemistry. The latter is a comprehensive work for the practitioner or teacher; the other a more compendious text-book for the student. We can, therefore, speak of them as a single work.

The late period at which we find time for a satisfactory examination of these valuable volumes, only enables us to echo the universal voice of the medical press as to their interest and intrinsic merit. In the rapid progress of this branch of research the writings of one year are replaced by the developments of the next, and even the laborious work of Simon, accurate and learned as it was, is entirely superseded by this.

It must, however, be a long time before we arrive at any settled conclusions in zoochemistry. The subject is so complex—its relations so numerous—that though we may solve the chemical actions of any given organic element out of the body, we have still left the problem of the influence of the vital force upon these reäctions—a problem which, ever variant, and passing through a long series of insensible gradations, must long remain unsettled.

Pure chemistry will never teach physiology. It is, of course, essential—without it we can do nothing; but it leaves after all the most necessary condition untouched. But those whose studies have not tended in this direction will be astonished to find how much of the problem of Life is solved, how far the persistent labors of scientific investigators have delved into the arcana of zoochemistry.

Lehmann is a practical experimentalist and observer. In the lesser of these works he has endeavored to present an epitome of the positive facts which may now be considered as the definite possessions of physiological chemistry. In the larger of them he goes more into the discussion of theories, and full debates of mooted questions. His position as a scientific laborer is so high as to carry with it an authoritative character, and nowhere will the student find so full and able a statement of our present knowledge in this department of science. We trust that it may meet that cordial encouragement from the profession which it so eminently deserves.

